# Capturing Patients’ and Clinicians’ Experiences of Using Video Consultations in Mental Health Outpatient Services: Qualitative Thematic Analysis

**DOI:** 10.2196/50580

**Published:** 2024-08-21

**Authors:** Ali Abbas Shaker, Erik Simonsen, Kristine Tarp, Radoslav Aleksandrov Borisov, John Aasted Sørensen, Henrik Bechmann, Stephen F Austin

**Affiliations:** 1 Psychiatric Research Unit Slagelse Denmark; 2 Department of Clinical Medicine University of Copenhagen Copenhagen Denmark; 3 Mental Health Services East Copenhagen University Hospital – Psychiatry Region Zealand Roskilde Denmark; 4 The National Research Centre for the Working Environment Copenhagen Denmark; 5 Mental Health Services South Copenhagen University Hospital – Psychiatry Region Zealand Maribo Denmark; 6 Research Unit: AI, Mathematics and Software Department of Engineering Technology and Didactics Technical University of Denmark Ballerup Denmark; 7 Institute of Psychology University of Copenhagen Copenhagen Denmark

**Keywords:** telemedicine, telepsychiatry, video consultation, mobile health, mHealth, COVID-19, synchronous technology

## Abstract

**Background:**

Over the last decade, there has been an increase in the evidence base supporting the efficacy of video consultations (VCs) in mental health services. Furthermore, the potential of VC treatment was also demonstrated during the COVID-19 pandemic. Despite these promising results and conducive conditions for VCs, several studies have highlighted that the uptake and implementation of VCs continues to be slow, even after the pandemic. To facilitate and strengthen the implementation of VCs and exploit their potential as a useful tool for mental health disorder treatment, there is a need for a deeper understanding of the issues and experiences of implementing and using VCs as a treatment modality in clinical practice.

**Objective:**

The aim of this study was to investigate patients’ and clinicians’ experiences and attitudes toward using VCs in clinical practice.

**Methods:**

Treatment was conducted through the VC modality. Semistructured interviews were conducted individually with patients (n=10) and focus group interview were conducted with clinicians (n=4). Patients had participated in weekly VC treatment over 2 months as part of mental health outpatient services in Denmark. Data from these interviews were analyzed using thematic analysis.

**Results:**

Thematic analysis of the patient interviews yielded two main themes: (1) adjusting to the practicalities of the VC format and (2) the practice of therapy using VCs. Patients experienced that using VCs was easy and convenient, and it was possible to establish and maintain a therapeutic alliance. They also described the contact as different to in-person therapy. The thematic analysis conducted on clinicians’ experiences of using VCs yielded three themes: (1) a shift in mindset from resistance to acceptance, (2) the contact is different when using the VC modality, and (3) adapting to a new way of working. Clinicians experienced that their initial concerns and resistance toward VC implementation gradually diminished over time as they gained clinical experience of using the modality. They expressed that contact with patients can be different when using the VC modality and that it took time to adjust to a new way of working therapeutically.

**Conclusions:**

Both patients and clinicians experienced that VCs could enhance access to treatment and be meaningfully integrated into clinical practice. In addition, both groups described the contact when using the VC modality as being different to in-person therapy. Future research could examine patients’ and clinicians’ perceived differences regarding contact when using the VC modality and the implications for therapeutic interventions.

## Introduction

### Background

It is estimated that in 2019, before the COVID-19 pandemic, approximately 970 million (13%) people globally were living with a mental health disorder, and most of these people did not have access to an effective mental health service [1]. Mental health services are challenged worldwide by the disparity between the limited number of mental health practitioners and the increasing number of patients needing mental health assessment or treatment [2]. With the growing aging population in Western countries and the increasing digitalization of people’s behavior, communication, and lifestyle, there is a need for user-friendly and meaningful technological solutions that can adapt to these changes [1,2].

Over the last decade, researchers worldwide have investigated video consultations (VCs) as an alternative mental health treatment modality, especially for rural patients. Qualitative studies have shown that VCs can increase access to care not only for patients who live in rural areas but also for patients who have problems with mobility, issues with access to transport, or economic limitations [3-8]. For patients with a busy daily life (eg, due to work or school or conflicting schedules), VCs can also be a preferred treatment modality because they can offer convenience and flexibility in the receipt of mental health treatment [7]. It has also been reported that VCs can reduce stigmatization because patients do not need to leave their homes for mental health services encounters [8]. Similar to the positive experiences reported in qualitative studies evaluating VCs, quantitative studies have also reported that mental health services delivered via VCs is equivalent to those delivered via in-person consultations [9-12].

Although there is a growing body of evidence indicating equivalence between VCs and in-person consultations, there are also several barriers to, and concerns regarding, the implementation of VCs in clinical practice. These barriers can generally be divided into three categories: (1) the technology itself, (2) the characteristics of the end users of the technology, and (3) the context in which the technology is implemented.

First, barriers related to the technology and VCs in particular involve concerns about the user-friendliness (ease of use) of the technology, the accessibility of the technology, confidentiality, and security features (eg, encryption, log-in and authentication, and software updates) [13-15]. Second, barriers linked to the users of the technology (patients and clinicians) include their physical and mental state when using the technology, their experience and technological skills in using the technology (digital competency), and the transition phase from in-person to VC modality. In addition, using VCs in clinical practice, particularly for therapeutic interventions, requires establishing a virtual therapeutic alliance, which can be experienced as new and challenging for some users [15,16]. Third, barriers related to the user context include whether VCs are used for general assessment, diagnosis, or treatment and the physical settings and environments where the VCs are implemented (eg, at the patient’s or clinician’s home, in a hospital, or in an outpatient service) [17-19]. Finally, the medical technology industry is in constant evolution, and new technological tools or improvements to existing systems are regularly introduced into the health care systems [20,21]. This potentially leads to a demand from both patients and clinicians to access and use cutting-edge technology in clinical practice but also requires ongoing training of users and an adjustment of clinical workflows to accommodate the new development.

Understanding the interactions between humans and technology is an important factor in determining how a digital solution will be received and used in clinical practice. Technology acceptance is defined as an individual’s intentional or voluntary use of a technology and is a core aspect of understanding the use of technology in the real world [22]. The technology acceptance model (TAM) describes how perceived usefulness and the ease of use of a technology impacts attitudes, the behavioral intention to use (intention), and actual use [23,24]. This theoretical framework can be used to understand how the attitudes and experiences of users (eg, clinicians and patients) may impact the use of VCs within clinical practice. Several reviews have shown that the TAM is a valid model to explain the use of technology in health settings [25]. This study explored patients’ and clinicians’ experiences when using VCs to provide insight into the factors that may impact its uptake or use in clinical practice.

### Objectives

The objective of this study was to investigate patients’ and clinicians’ experiences and attitudes toward using VCs in clinical practice. Understanding the perspectives of patients (service users) and clinicians (service providers) is key to the successful implementation of VCs in treatment. Developing a deeper understanding of the users’ experiences of VCs could help generate ideas to facilitate and strengthen the implementation of VCs and exploit their full potential as a tool for the treatment of mental health disorders.

## Methods

### Study Design

A qualitative study design with an exploratory approach was used to investigate patients’ and clinicians’ experiences of using VCs in clinical practice. The COREQ (Consolidated Criteria for Reporting Qualitative Research) guidelines, which aim to enhance the transparency of conducting and reporting qualitative research, were followed to report this study [26]. The COREQ guidelines encompass various aspects of the study, including study design and procedure, data collection and analysis, and the reporting of the study in a structured manner. This qualitative study was part of a larger study that collected quantitative data about how clinicians used VCs in clinical practice; the results have been published elsewhere [27].

### Settings and Participants

The study was conducted at a mental health outpatient service in the southern part of Region Zealand, Denmark. The clinic annually treats approximately 550 adult patients (aged >18 y) with anxiety, posttraumatic stress disorder (PTSD), depression, and personality disorders, both individually and in group-based settings. Nearly 50% of the patients are aged between 18 and 29 years. During the COVID-19 pandemic, the recommendations from the health ministry were to conduct VCs with patients, but in-person consultations were still an option for patients. The VC system was available before the pandemic but not used sufficiently in clinical practice.

A convenience sample of patients and clinicians who used VCs in the treatment of nonpsychotic disorders were invited to participate in interviews. Participants were recruited from an implementation study conducted at the same clinical site that collected data on a range of quantitative outcomes such as acceptance, demand, negative outcomes, and therapists’ reported focus for patients receiving treatment via VCs (n=15) or in person (n=19). A detailed description of the design and results of this study is contained in another publication [27].

Patients were eligible to participate in the study if they were aged >18 years, were newly diagnosed with a nonpsychotic disorder, and owned a smartphone or a computer capable of supporting VCs. Clinicians had an allied health background (eg, psychologist, psychiatric nurse, or psychiatrist); therapeutic education in cognitive behavioral therapy, mentalization-based therapy, schema-focused therapy, or dialectical behavioral therapy; and a minimum of 1 year of clinical and therapeutic experience.

### VC Modality

Patients received diagnosis-specific treatment based on their needs, and treatment was conducted through the VC modality. This treatment typically consisted of 8 weekly sessions over a period of 2 months and usually included a combination of psychoeducation, psychotherapy, support, and medication. The focus of psychotherapy was on promoting a better understanding of the difficulties experienced, possible triggers, and the development of appropriate strategies to deal with these difficulties. Patients receiving treatment via VCs had indicated that they were open to participating in treatment via this modality. The participants (patients and clinicians) in the study had little or no in-person interactions before commencing treatment via VCs.

### Procedure

Patients and clinicians who had participated in the 2-month treatment course via VCs were invited to participate in interviews. The interviews in the study included individual semistructured interviews with the patients and a focus group interview with the clinicians. An interview guide [28] was developed to facilitate the semistructured interviews with the patients, while a discussion guide [29] was developed for the focus group interview with the clinicians. Both guides consisted of open-ended questions and probes to cover the important aspects of using VCs in clinical practice. The guides were developed by researchers with experience in telemedicine research. The guides included (1) an introduction section that clarified the purpose of the interviews, followed by (2) general questions related to the experience of using VCs, (3) questions regarding the use of VCs for clinical treatment, (4) probes regarding any perceived differences between VCs and in-person consultations as well as (5) probes regarding suggestions for changes or improvements regarding the use of VCs in clinical practice, and (6) questions about other experiences and reflections about VCs not already covered in the interview. The researcher conducting the interviews had no prior working relationship with the participants.

The semistructured interviews were conducted virtually or in person at the patients’ homes or at the mental health outpatient service. Patients were asked open-ended and probing questions, and the interviewer (AAS) was aware and reflective of his own beliefs regarding VCs. The interview guide was designed to allow participants ample time to elaborate on their own perceptions of VCs for each of the topics covered in the interview guide. The interviews ranged from 15 to 45 minutes and were audio recorded.

The focus group interview was held at the end of the study at the mental health outpatient service where the clinicians (psychiatrists, psychiatric nurses, and psychologists) conducted their daily clinical work. The first author (AAS) mediated the focus group interview and facilitated discussion and dialogue between the participants while keeping the focus group interview on track. The interview lasted 1 hour and was audio recorded.

### Data Analyses

The study took an inductive approach to collect information about patients’ and clinicians’ experiences of using VCs as a treatment modality. Thematic analysis was used as a methodology to analyze the data generated during the interviews following the guidelines outlined by Braun and Clarke [30]. The analysis aimed at identifying broader patterns of latent meanings by transcending the descriptive level of the data. A guiding principle was the “keyness” of the theme in regard to its ability to capture information important to the research focus [30,31].

The analytic procedure entailed (1) becoming familiar with the data, which involved reading and rereading the data set and writing memos; and (2) generating initial codes, where data were labeled and organized into meaningful groups. Codes were organized around central ideas or concepts (3) searching for themes (defining different theme properties), (4) reviewing tentative themes (collapsing overlapping themes and reworking and refining codes and themes), (5) labeling themes (fitting the broader data set to respond to the research questions), and (6) summarizing the data. The themes were conceptualized as patterns of shared meaning, cohering around a central concept—the central idea or meaning.

The primary author (AAS), who is a qualified IT engineer and medical doctor, conducted the thematic analysis in consultation with the last author (SFA) to help construct codes and themes through an iterative process. The third author (KT), an expert in qualitative analysis, provided overall guidance and supervision of the analysis. Two separate thematic analyses were conducted, one covering the patients’ perspectives and the other covering the clinicians’ perspectives of using VCs in clinical practice.

The thematic analysis emphasized the importance of the researchers’ subjectivity as an analytic resource and their reflexive engagement with theory, data, and interpretation. Different strategies were used to increase trustworthiness and promote reflexivity during the analysis [32,33]. First, all interviews were recorded, transcribed verbatim, and uploaded to a secure central archive so that all coders could have prolonged engagement with the raw data and thereby improve the credibility of the analysis. Second, raw data were transferred to NVivo 12 Pro (Lumivero) so that each step of the analysis could be documented. This created an audit trail and promoted confirmability. Third, the 2 researchers (AAS and SFA) who conducted the thematic analysis engaged in peer consultation to promote triangulation regarding the main themes. Furthermore, a third author (KT) was consulted in the peer consultation to clarify themes that could not be resolved between the 2 main authors. Finally, the results from the thematic analysis were presented to the entire research team, and their reflections and ideas were incorporated into the analysis where deemed appropriate.

### Ethical Considerations

All participants received verbal and written information about the research project before providing informed consent. All material from the interviews was anonymized and stored on a secure server. Approval for the study was obtained from the Scientific Ethics Committee for the Region of Zealand (EMN-2021-00019), and from the institutional review board of the Region of Zealand (REG-003-2021).

## Results

### Patient Interviews

#### Thematic Analysis

A total of 10 patients who received treatment via VCs were interviewed. Participants’ ages ranged from 18 to 40 (mode 29) years. The majority (7/10, 70%) were female. The participants either had a diagnosis of an anxiety disorder (5/10, 50%) or a borderline personality disorder (5/10, 50%). Patients who participated in the semistructured interviews were considered representative of the typical referrals to the clinic because >80% (34/40) of those invited agreed to participate in the study [27].

**Figure 1:**
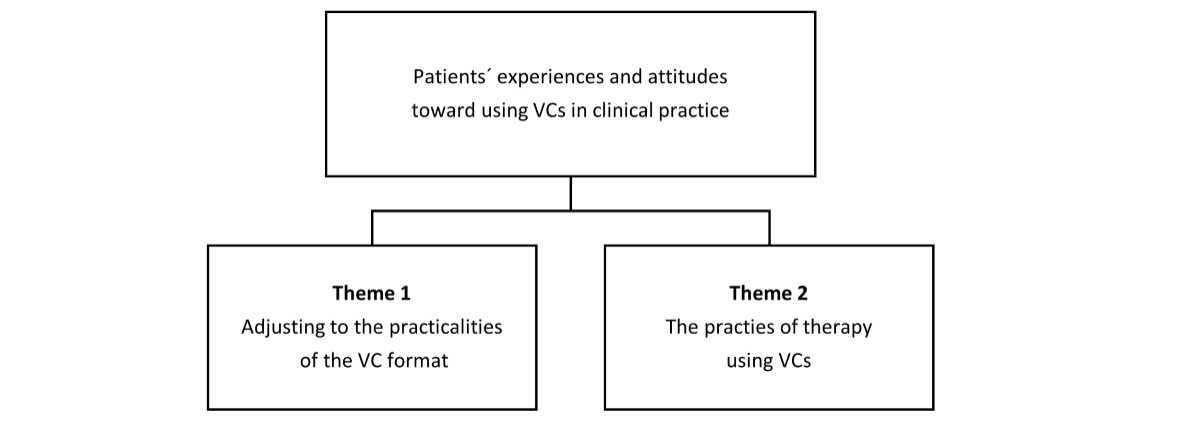
Thematic tree of patients’ experiences of using video consultations (VCs).

The thematic analysis conducted on the 10 semistructured interviews yielded 2 themes (Figure 1): (1) adjusting to the practicalities of the VC format and (2) the practice of therapy using VCs.

#### Theme 1: Adjusting to the Practicalities of the VC Format

This theme describes how patients experienced the practical aspects concerning treatment conducted through VCs. The theme’s focus was generally characterized by the patients’ experiences and descriptions, outlining that VCs are a practical tool where they could participate in psychiatric treatment without disruption to their daily lives and routines. VCs were perceived as particularly advantageous for patients who have a busy everyday life with school or work. Some of the patients expressed that they probably would have had to cancel their treatment appointments if VCs were not an option:

Well, I don’t think we could have appointments that often. Especially because my school schedule is fluctuating, so it’s quite possible that I’ll have to cancel an appointment or two. Or many.

P1

The theme covered other positive aspects highlighted by participants, such as reduced travel time, related travel costs, and stress. The patients experienced that they could sit at home without spending time on making practical preparations for treatment (ie, arranging transportation, getting dressed, and taking time off from school or work). VCs were experienced as a valuable and stress-reducing treatment modality that eliminates all these “unnecessary” preparations and practicalities:

I think it has saved me a lot. I don’t have to spend a lot of energy on traveling back and forth. And during the weeks when it has been tough and I’ve been extra tired, I could just do it over video, so in many ways, it has made it easier to do it over video. There were often times when I thought, “I have a meeting tomorrow,” ah, it is nice that it’s just over video.

P7

Both quotes highlight the advantages of VCs as perceived by patients because the treatment modality allowed greater flexibility, accommodating the demands of everyday life. Participants described this flexibility as promoting attendance.

However, the patients also described that they sometimes experienced technical challenges associated with VC technology. These challenges were generally related to poor sound and image quality, but these practical experiences related to the technology did not significantly detract from the overall positive patient experience regarding using VCs in clinical practice. When technical challenges arose during the appointments, the patients and clinicians tried to collaborate to solve them:

Well, you know, it’s fantastic when it works. But the problems lie with the equipment the government has purchased. It has caused clinicians to make some phone calls. Three times she [clinician] sounded like a mouse. Haha. Then we figured out that I needed to switch devices. I also have a tablet. And it was the same issue there. Then she switched to a computer, and it worked. I mean, I have to say, what I had was so poor. Um, what’s it called. Having a headset helped a lot. What they’re using doesn’t work optimally, but they still use it. But the headset works just fine.

P5

This quote highlights how relatively minor technical issues could impact the quality of communication during VCs and the need for both parties to be tolerant and flexible in finding a solution*.*

#### Theme 2: The Practice of Therapy Using VCs

The second theme that was constructed from the patients’ interviews covers how their experiences of using VCs affected establishing and maintaining therapeutic engagement and alliance. The patients expressed that they were less affected by their anxiety symptoms when the appointment was conducted virtually, making it easier for them to relax and establish or maintain engagement in therapy:

But it’s also because I have no opportunity, I mean, regarding my anxiety, to go anywhere because it’s so severe. So I think it was really good that they could do this video thing. Otherwise, I wouldn’t have received any treatment.

P3

The patients also experienced that VCs contributed to reducing stigma because the modality promoted access to psychiatric treatment, giving them the possibility to talk about their mental health problems with a therapist without having to visit the outpatient service in person. The ability to access treatment was seen as the first step to establish meaningful engagement with psychiatric services:

Well, also the fact that I could have the option to do it over video meant a lot to me, actually, because in the beginning, I was incredibly anxious about going to the psychiatric facility. I immediately thought I would be locked up and had all sorts of anxious thoughts. So I was really grateful for the opportunity to do it over video.

P2

Both quotes describe how treatment via the VC modality was perceived to promote engagement in treatment by reducing anxiety compared to in-person treatment.

Regarding the therapeutic alliance, participants expressed that it could be established and maintained when treatment was conducted virtually. However, this alliance or contact with the therapist was considered different and for some of the participants was not considered to be at par with in-person therapy:

If we see it as a spectrum, then what’s it called, being there in person would be 100% and video would be like 60-75%. But sometimes you need that 100% of being physically present. You can look at each other and see that she’s there, but it’s not the same as sitting in the same room.

P6

In addition, while patients described that therapeutic alliance can be maintained sufficiently over video, there were also some challenges. These challenges to therapeutic alliance were often described when patients experienced powerful emotions or when dealing with complex and sensitive issues:

Yes, that’s if I were to become very emotional, if I were to get very upset. In those moments, it would have been nice to have someone in person to meet with. Because I know that if I start having anxiety attacks, I need someone to put their hand on my shoulder and give me a hug, which can’t really be done through a video consultation.

P7

While patients acknowledged that meaningful alliance could be achieved over VCs, they were perceived to have a different quality. In addition, contact when using the VC modality was described as insufficient when patients experienced extreme emotions and wanted some physical comforting.

In summary, the patients experienced VCs as a convenient tool, which could easily be integrated into their daily lives. The convenience of using the technology facilitated and contributed to increased engagement with the treatment, although the participants also experienced a difference in contact when using VCs compared to using in-person therapy.

### Clinician Focus Group Interview

#### Thematic Analysis

A total of 4 clinicians (nurses: n=3, 75%; psychologist: n=1, 25%) participated in the focus group interview. They had an average of 10 years of experience treating patients with anxiety, depressive symptoms, and personality disorders using standard in-person treatment. Over the course of the study, each clinician conducted on average 26 VCs. Thus, these clinicians gained experience and familiarity with the video technology across several patient treatment sessions.

**Figure 2:**
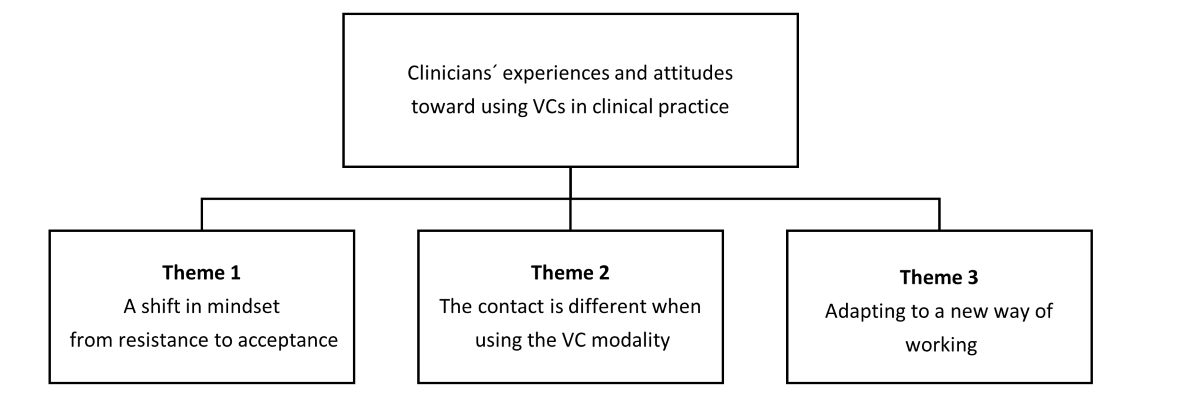
Thematic tree of clinicians’ experiences of using video consultations (VCs).

The thematic analysis conducted on the focus group interview yielded three themes (Figure 2): (1) a shift in mindset from resistance to acceptance, (2) the contact is different when using the VC modality, and (3) adapting to a new way of working.

#### Theme 1: A Shift in Mindset From Resistance to Acceptance

This theme was characterized by the clinicians experiencing a shift in their attitude toward using VCs. At the beginning of the study, the clinicians described a perceived resistance to using VCs in clinical practice; however, as they gained experience with VCs, they developed a new and more nuanced attitude toward using VCs in clinical practice. They described a range of reasons for their initial resistance. First, the clinicians were worried that their professional identity would disappear when the treatment took place over VCs:

That it is just going to be robots, hahaha, and video, and there will be no human interaction with the mentally vulnerable people, and I don’t like that...but I had a different experience along the way.

C1

Second, their resistance was also associated with concerns about whether VCs would technically work and whether they possessed the technical skills to conduct VCs:

I thought...phew, I’m not very tech savvy.... I didn’t grow up with this technology, so I found it a bit scary and a bit intimidating, but I also thought it was the future.

C4

Third, resistance was also associated with clinicians’ worries that VCs would be less effective for patients than in-person consultation:

I had a bit of a prejudice that there was a difference and that it would lean more towards the advantage of in-person [consultation] rather than video. That was my prejudice.... I actually felt a bit embarrassed about it.

C2

However, over time, the initial mindset shifted from resistance to a more accepting attitude, and clinicians’ worries diminished as they continued using VCs in clinical practice:

If I suggest video to the patients, they become ecstatically happy. Before this project, I would never, ever have suggested video instead. But I do now. I do it a lot now. My prejudices have disappeared.

C2

My attitude has absolutely changed. Now I know it, I have tried it, and it makes so much sense for those patients who cannot leave their homes.

C3

The clinicians’ initial concerns—rooted in their professional identity as well as doubts about their technical abilities and the perceived effectiveness of treatment via VCs—gradually changed due to their experiences of using VCs in clinical practice.

#### Theme 2: The Contact Is Different When Using the VC Modality

The clinicians experienced that the contact via VCs is different compared to in-person contact; for example, it was perceived that the contact when using the VC modality was artificial compared to using in-person consultations; and clinicians compensated for, or tried to reduce, this feeling of “artificiality” by creating new routines:

You could also say, “Have you had a cup of coffee?” over video or “Are you going to get yourself a cup of coffee?” or “I’m going to go get myself a coffee now.” But to make it less artificial, you could say something like that. Or something else. Just to lighten it up for myself, you know.

C2

In addition, the clinicians felt that not all consultations were suitable to be conducted over video. They described that they were more reluctant to discuss sensitive and complex topics with the patient that may induce emotional reactions:

Well...now I have many borderline patients, and I feel...it’s not easy because there are a lot of emotions involved. Sometimes they agree to video sessions. But then they say, “Oh, we can’t do it after all.” So, I think it depends a lot on the patient group.

C1

The clinicians also felt that contact with patients with poor appointment adherence could be strengthened by offering them VCs rather than terminating the patients’ affiliation with the clinic.

Also, if they cancel...then you can be cheeky: “Okay, what is it about? Should we change it to video calls instead if it’s better?” And there are some who say yes to that.... So, we avoid some of those no-shows.

C3

The quotes highlight that clinicians clearly experienced that the contact when using the VC modality was different and that they were creative in coming up with new ways to build an alliance. Conversely, they also voiced a concern about their ability to deal with complex emotions when using VCs.

#### Theme 3: Adapting to a New Way of Working

This theme describes how the clinicians perceived using VCs as a new way of working and how VCs can be effectively integrated into a busy mental health outpatient service. The clinicians described that they needed time, training, and experience with the technology before it could be implemented successfully in clinical practice. They also recognized that the introduction of VCs as a tool within treatment could produce some tension. However, the clinicians described that being open and transparent with the patients about the fact that using VCs is a new way of working was an important factor in easing the implementation of VCs in practice:

For many, VC [video consultation] is new, just like it is for us...so, we have to address it...and maybe tell that both of us [patients and clinicians] are tense in this situation.

C4

The clinicians also described that while the COVID-19 pandemic helped facilitate the implementation of VC, it also caused stress because they experienced that implementation took place too quickly. They described that, in the future, a smoother transition at a more measured pace was necessary when new technology was introduced into clinical practice. Nevertheless, participating in the study and gaining experience with providing virtual treatment made the clinicians more confident in using VCs in clinical practice:

I feel more comfortable now than in the beginning. I am more comfortable.... And I can offer it to those who have difficulty leaving their homes. And I do it in an easier way than before.

C2

Overall, clinicians initially experienced worries about whether VCs could be used in clinical practice. These perceived concerns faded as they became familiar with VC technology and adapted to providing treatment using this modality in clinical settings. Clinicians described that there was a need for a maturation period to build up further experience with VCs before they could be integrated into their daily working routines. Finally, there was a clear perception that the contact was different when using the VC modality, and this could potentially have clinical implications, such as a reluctance to work with patients with complex emotional issues.

## Discussion

### Principal Findings

This qualitative study investigated patients’ and clinicians’ experiences of using VCs in a mental health outpatient service. The findings indicated that VCs could be meaningfully integrated into mental health services. Themes constructed from patient and clinician interviews highlighted various issues that could impact the utility and acceptance of VCs as a modality for providing mental health treatment.

There were a number of commonalities across the themes for patients and clinicians. First, both groups perceived that VCs contributed to facilitating access to, and provision of, treatment, especially for patients with a busy everyday life with school or work, as well as those unable to receive in-person treatment due to travel distances or the constraints imposed by their mental health disorders. Ashwick et al [7], who investigated the use of VCs for patients with PTSD in a qualitative study, found that VCs increased patient attendance and engagement with the virtual treatment, consistent with this study. The authors also described that the patients experienced a sense of “personal accountability” for maintaining attendance and engagement in the video treatment. In addition, patients experienced that finding a private room free from the presence of partners and children could be challenging, and it was easier to postpone or not attend the virtual meeting at all if they did not have the energy to participate in the VC [7]. Patients in this study did not describe experiencing a sense of “personal accountability” for maintaining attendance and engagement in VCs, although there were no patients with PTSD included in this study. The scientific literature indicates that patients with PTSD can have low treatment adherence, with 35% to 63% failing to complete a treatment course [34]. However, future studies should investigate how virtual treatment engagement can be maintained and increased over time and space.

A second common theme for both patients and clinicians was that the perceived contact was different when using the VC modality compared to using in-person consultation. Clinicians described a number of practical ways in which they attempted to create this bond or good contact with their patients despite this perceived difference with in-person treatment. They also described different ways to make VCs more “real or authentic” and potentially compensate for the fact that treatment was being conducted over video. Examples of these adjustments included asking patients virtually “if they have had a cup of coffee” at the start of a session or suggesting that patients have an object (eg, a cushion or something they could hold onto) with them that could help soothe them if they became upset. This “practice-orientated” mindset is an interesting finding and could be seen as compensatory behavior in response to treatment that is conducted virtually.

Both groups also described the perceived limitations of, or reservations regarding, conducting treatment using the VC modality in relation to discussing complex or sensitive issues, which could trigger strong emotional reactions. Several studies have reported similar findings that it can be challenging to address sensitive subjects that arise during psychotherapeutic treatment over video and described VCs as being superficial [13,17,18]. Interestingly, despite this perceived limitation of VCs, many efficacy studies show that VCs are as effective as in-person consultations [9-12]. Future studies should address how in-depth consultations and discussions of complex subjects can be conducted virtually and what potential positive or negative effects this may have in clinical practice.

Clinicians described being initially resistant to the idea of using VCs for psychiatric treatment, but this resistance diminished once they gained experience with VCs. This change in attitude among clinicians has been found in a number of studies examining the implementation of VCs [3,17]. Clinician attitudes are central to the uptake of VCs in clinical practice because they are seen as “gatekeepers” who determine whether VCs are offered to patients [16]. Thus, it is important to make these clinicians’ experiences of using VCs accessible to all clinicians when considering the adoption of VCs.

Patients largely held more positive views on VCs than clinicians, but they did describe that technical issues could be disruptive when receiving treatment. Some patients reported instances where the screen froze, and sometimes the sound was lacking. However, this was not a regular problem and did not affect the generally positive attitude of patients toward using VCs in clinical settings. This finding is consistent with other studies showing that as long as the technical challenges are not persistent, patients tend to perceive them as less significant [13,14].

An interesting theme from the clinician focus group interview was that the VC format introduced a new way of working and brought significant change to their clinical practice. VC implementation and uptake need maturation time, allowing clinicians to develop routines and gain experience before VCs can be fully integrated into their clinical work. Thus, although the clinicians expressed that their mindset changed from “resistance to acceptance,” there is still a need for maturation with regard to the transition from “accepting to implementing” the technology in clinical practice. A recently published guide from the World Health Organization and a comprehensive systematic review recommend several interventions to strengthen the video implementation, including training and guidance for end users, technical support for end users, and continuous evaluations and improvements in workflows and technical solutions related to the application of the video system in mental health care settings [35,36].

Using the TAM [23] to frame the patients’ and clinicians’ experiences of using VCs allowed us to identify a number of common themes that were consistent with aspects of the TAM that covered the ease of use, perceived usefulness, and the acceptance of VCs as a treatment modality for mental health problems. Common themes that highlighted the advantages of VC (flexibility as well as saving time and energy) could be seen as increasing acceptance and use, while common themes that concerned the limitations or challenges with VCs (technical issues, difficulty discussing complex and sensitive issues, and concerns about adequate technology literacy) may reduce VC acceptance and use in clinics.

Given that quantitative data about the use of VCs in clinical practice was also collected from these clinicians as part of another study [27], it was possible to explore the link between clinician experiences collected from the focus group interviews and how VCs were actually used in practice. Self-reported data from these clinicians showed that VCs were primarily used for a shorter time with a focus on supportive counseling (80% of the sessions) compared to in-person sessions that were significantly longer and had a much greater psychotherapy focus [27]. Thus, framing these outcomes using the TAM, it is possible to hypothesize that clinician attitudes concerning the limited usefulness of VCs when used with patients with complex emotional issues directly impacted the acceptance and use of VCs in clinical practice (eg, VCs were primarily used for supportive counseling rather than psychotherapy).

### Strengths and Limitations

One of the strengths of the study is that it captured patients’ and clinicians’ perspectives on using VCs within the same mental health outpatient service. Analyzing experiences from treatment dyads generated a more holistic understanding of the VC experience. In addition, interviewing both clinicians and patients allowed the exploration of commonalities and differences in the 2 user perspectives. A second strength is that the study included people with a range of mental health disorders from a clinical setting that included a range of mental health professionals. Thus, the participants were considered representative of a clinical setting, which may increase the ecological validity and generalizability of the results to other clinical settings.

The study also has several limitations. First, it included a self-selected convenience sample, consisting of participants who were willing to receive treatment via VCs. This sample may be considered biased because these individuals might have a more positive or open attitude toward using VCs, which in turn could be reflected in the results. Second, the sample sizes for patients and clinicians were very small, potentially limiting the generalizability of the results to the clinical setting. In addition, the study was conducted within a specific mental health outpatient service in Denmark, which also limits the generalizability of the findings to broader populations and different health care systems. Third, a deeper understanding of the clinicians’ experiences might have been achieved if individual semistructured interviews had been conducted rather than a focus group. Fourth, the experiences collected were based on a relatively short intervention (2 months), which made it difficult to understand how experiences of using VCs may develop or change over time. Fifth, it is important to acknowledge that the background and experience with VCs of the primary researcher (AAS) may have influenced the interviews and subsequent analysis of qualitative data. As thematic analysis involves the construction of themes grounded in qualitative data, various strategies were used to promote trustworthiness in the analysis and themes constructed. Finally, the study was conducted during the COVID-19 pandemic, which may have impacted both patients’ and clinicians’ attitudes and behaviors regarding VCs.

### Implications for Clinical Practice and Research

This study has several important practical implications. First, the results from the study show that VCs can be meaningfully implemented in mental health services, and treatment via this modality can contribute to establishing and maintaining therapeutic engagement and alliance. This finding is especially relevant for patients who, for various reasons, find it difficult to attend appointments in person. Second, this study has demonstrated that clinicians are accepting of, and open to, the use of VCs in clinical practice; however, this acceptance may require time to develop and mature because clinicians need to acquire the experiences and skills necessary to incorporate VCs into clinical practice.

Interestingly, both patients and clinicians experienced that the contact when using the VC modality was different from in-person contact, and in some cases, it was described as being “superficial and artificial.” Both groups described the perceived challenges of using VCs in the treatment of patients with complex emotional issues. Future studies need to investigate the impact of this perceived different contact in VCs and its implications for the application and adaptation of therapeutic techniques in psychiatric treatment. Furthermore, one could move beyond the traditional understanding of VCs and investigate how cutting-edge technologies, such as artificial intelligence as well as augmented and virtual reality telepsychiatry can reduce the “artificial experience” of VCs and enhance telepresence, creating a new treatment environment where complex treatment issues can be meaningfully addressed and discussed.

### Conclusions

From the patients’ perspective, VCs were a convenient tool that could be integrated into their daily lives and promote treatment adherence. While clinicians experienced initial concerns and resistance toward VC implementation, these reservations diminished over time as they gained clinical experience of using the VC modality. Future studies could examine strategies to influence user attitudes and the acceptance of VCs, particularly regarding their perceived limitations, which may directly impact the implementation and use of VCs within mental health services.

## Abbreviations

COREQ: Consolidated Criteria for Reporting Qualitative Research
PTSD: posttraumatic stress disorder
TAM: technology acceptance model
VC: video consultation

## Data Availability

The data sets generated during and analyzed during this study are available from the corresponding author on reasonable request.
